# Basal Cell Carcinoma of the Umbilicus: A Comprehensive Literature Review

**DOI:** 10.7759/cureus.770

**Published:** 2016-09-07

**Authors:** Saisindhu Narala, Philip R Cohen

**Affiliations:** 1 Department of Dermatology, University of California, San Diego

**Keywords:** basal cell carcinoma, umbilicus

## Abstract

Basal cell carcinoma (BCC) typically occurs in sun-exposed sites. Only 16 individuals with umbilical BCC have been described in the literature, and the characteristics of patients with umbilical BCC are summarized. PubMed was used to search the following terms: abdomen, basal cell carcinoma, basal cell nevus syndrome, and umbilicus. Papers with these terms and references cited within these papers were reviewed. BCC of the umbilicus has been reported in five men and 11 women; one man had two tumors. Two patients had basal cell nevus syndrome (BCNS). Other risk factors for BCC were absent. The tumor most commonly demonstrated nodular histology (64%, 9/14); superficial and fibroepithelioma of Pinkus variants were noted in three and two patients, respectively. The tumor was pigmented in eight individuals. Treatment was conventional surgical excision (87%, 13/15) or Mohs micrographic surgery (13%, 2/15); either adjuvant laser ablation or radiotherapy was performed in two patients. The prognosis after treatment was excellent with no recurrence or metastasis (100%, 16/16). In conclusion, BCC of the umbilicus is rare. It usually presents as a tumor with a non-aggressive histologic subtype in an individual with no risk factors for this malignancy. There has been no recurrence or metastasis following excision of the cancer.

## Introduction and background

Basal cell carcinoma (BCC) is the most common type of skin cancer. Although it is rarely fatal, BCC can cause significant morbidity due to local invasion. Ultraviolet exposure is the primary risk factor associated with this malignancy, and therefore, BCCs typically arise on sun-exposed areas [1]. Albeit less commonly, BCCs can arise on sun-sheltered sites such as the axilla, breast, buttock, foot, groin, mouth, nail bed, nipple and areola complex, palm, penis, scrotum, and vulva [2-6]. The characteristics of patients with umbilical BCC, including the woman described in this report, are summarized.             

### Illustrative case

**Figure 1:**
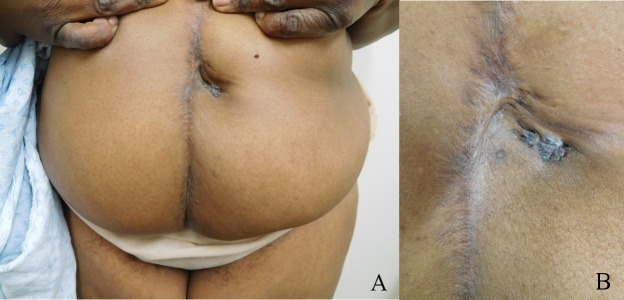
Umbilical basal cell carcinoma (BCC) presenting as a pigmented plaque Distant (A) and closer (B) views of an umbilical BCC presenting as a pigmented plaque measuring 3.0 x 1.5 cm on the umbilicus of a 50-year-old, Fitzpatrick skin type 5, African-American woman who presented for routine skin examination.

A 50-year-old Fitzpatrick skin type 5, African-American woman presented for a routine skin examination. She had no prior exposure to arsenic or environmental toxins or ionizing radiation. The patient has basal cell nevus syndrome with multiple previous BCCs; her mother also had pits on the palms and soles and clinodactyly. She had no prior melanoma or squamous cell carcinoma. The umbilical BCC presented as a pigmented plaque measuring 3.0 x 1.5 cm on the umbilicus and was located adjacent to a mid-abdominal surgical scar from ventral hernia repair with mesh placement four months prior (Figures 1A, 1B). She had a history of several surgeries at the site. 

**Figure 2:**
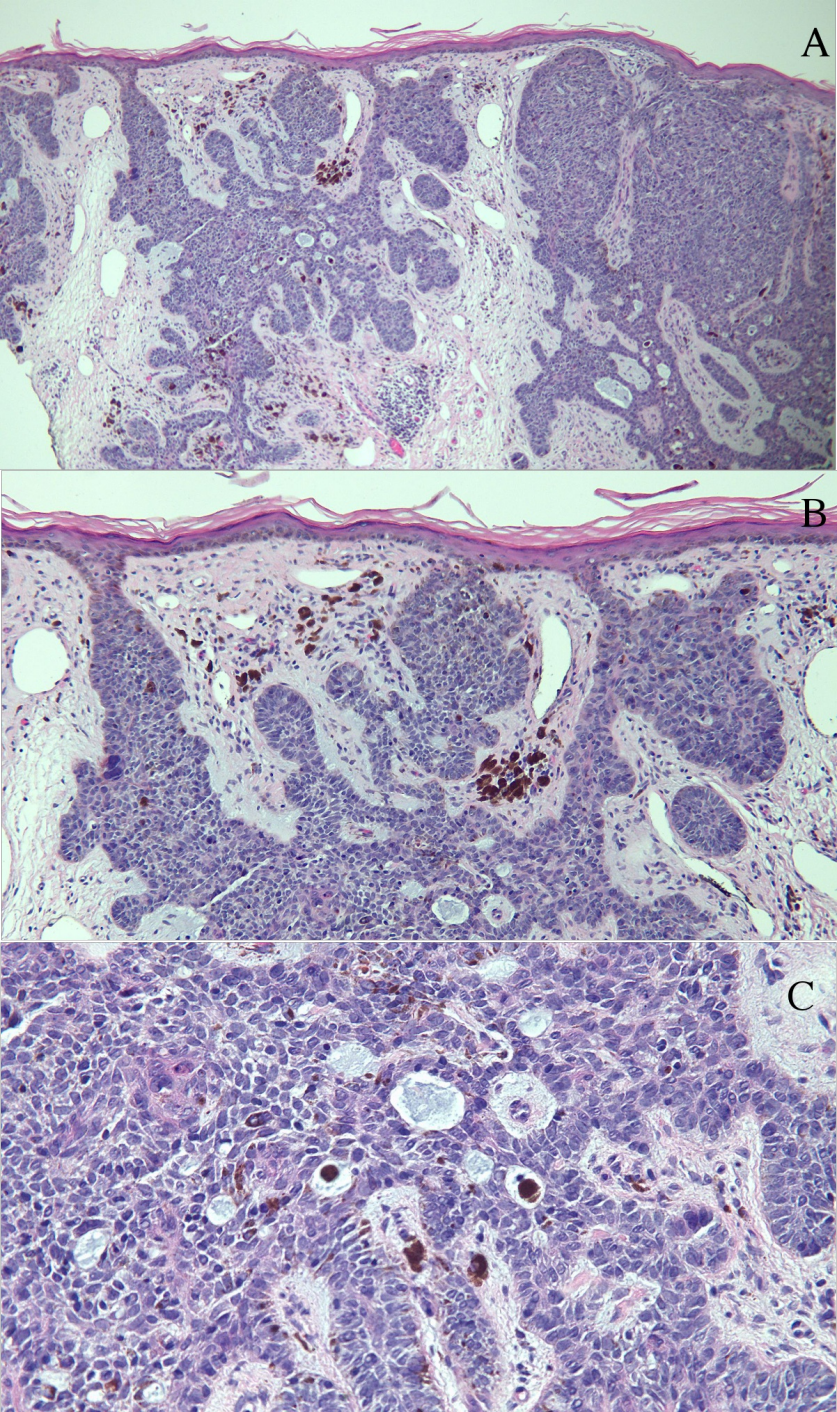
Umbilicus punch biopsy histology Low (A) and higher (B and C) magnification views of a 3 mm punch biopsy of the umbilical plaque in the woman from Figure 1. Microscopic examination showed nodular aggregates of basaloid tumor cells extending from the epidermis into the dermis (A). In the tumor and surrounding stroma, there were deposits of melanin, some of which were present in melanophages (B and C). Mucin, with or without melanin-containing melanophages, is present within the tumor aggregates (C). (hematoxylin and eosin stain: a = x4, b = x10, c = x40).

Punch biopsy of the umbilical plaque revealed nodular aggregates of basaloid tumor cells extending from the epidermis into the dermis (Figure 2A). In the tumor and surrounding stroma, there were deposits of melanin, some of which were present in melanophages (Figures 2B, 2C). Mucin, with or without melanin-containing melanophages, was present within the tumor aggregates (Figure 2C). These findings established the diagnosis of a pigmented BCC. Given the proximity of the BCC to the patient’s previous ventral hernia surgery incision, she was referred to a surgical oncologist for excision of the residual tumor.

## Review

### Epidemiology

Steck and Helwig reported the first cases of umbilical BCC in 1976. They wrote a review of 112 umbilical tumors collected at the Armed Forces Institute of Pathology, two of which were BCCs. Interestingly, one of the tumors was associated with an epidermal inclusion cyst [9]. Subsequently, only a small number of individual reports have been published. We summarized the characteristics of 16 patients with a total of 17 umbilical basal cell carcinomas (Table 1) [7-18].   

**Table 1:** Characteristics of Patients with Umbilical Basal Cell Carcinoma Abbreviations: AA = African-American; BCC = basal cell carcinoma; BCNS = basal cell nevus syndrome; Bx = biopsy; Ca = Caucasian; CID = cutaneous immunocompromised district; Clin = clinical diagnosis; Cm = centimeter; CO_2_ = carbon dioxide; CR = current report; Dx = diagnosis; FeP = fibroepithelioma of Pinkus; F/U = follow-up; Hx = history; LA = laser ablation; MMS = Mohs micrographic surgery; Mos = months; Path = pathologic diagnosis; PS = prior scar; NR = no recurrence; NS = not stated; Ref = reference; SCC = squamous cell carcinoma; TP - treatment pending; UP = ultrapulse ^a^The patient had an epidermal inclusion cyst at the site of the tumor. ^b^Two independent umbilical FeP tumors arose in one patient. They shared similar features except that one of the tumors was pigmented. ^c^The patient had a ventral hernia repair at the site. ^d^The patient had a scar from a prior laparoscopic port at the site.

Case	Age, Race, Sex	Risk and/or related factor	Present before dx (mos.)	Pigment	Histologic type	Size (cm)	Treatment	F/U (mos., result)	Ref
1	21 M	NS	NS	NS	Nodular	NS	Surgery	48, NR	7
2	48 M	BCNS	NS	NS	Nodular, Superficial	6.5x4.5	Surgery, UP CO_2_ LA	15, NR	8
3	52 M	CID^a^	NS	Path	Pigmented, Superficial	NS	Surgery	NS	9
4	54 M	NS	12	Clin	Nodular	4.0x3.0	Surgery	52, NR	10
5^b^	83 M	Hx SCC, BCC	84	Clin, Path	FeP, Pigmented,	0.1x0.1	NS	NS	11
6	27 Ca F	NS	24	NS	Nodular	0.4	Surgery	NS	12
7	43 F	NS	240	NS	Superficial	2.7x1.5	MMS	NS	13
8	50 AA F	BCNS, PS^c^	36	Clin, Path	Nodular, Pigmented,	3.0x1.5	Surgery	TP	CR
9	54 F	PS^d^	24	NS	Nodular	2.5	Surgery	12, NR	14
10	67 F	NS	3	Clin	FeP	NS	Surgery	NS	15
11	68 F	NS	6	Clin	Nodular	1.8x1.5	Surgery	24, NR	10
12	71 F	NS	NS	NS	NS	NS	Surgery	NS	16
13	75 F	NS	12	NS	NS	NS	Surgery	NS	17
14	76 F	NS	NS	NS	NS	NS	Surgery	NS	9
15	80 Ca F	NS	24	Clin	Nodular	2.0x1.8	MMS, radiation	NS	18
16	94 F	NS	12	Clin	Nodular	2.0x2.0	Surgery	12, NR	10

Umbilical BCCs were described in five men (31%) and 11 women (69%) (men: women = 1: 2.2). The patients ranged in age from 21 to 94 years, with a median age of 61 years at the time of diagnosis. The men ranged from 21 to 83 years of age (median = 52 years), and the women ranged from 27 to 94 years of age (median = 67.5 years). The majority of men were diagnosed before their sixth decade (80%, 4/5) while the majority of women were diagnosed during or after their sixth decade (60%, 6/10). The race was not specified for most of the patients with umbilical tumors. Two patients were Caucasian and one patient was African-American. Two patients with no reported race were described as having fair skin (Cases 1 and 7).

### Clinical presentation

Many of the tumors were diagnosed after a change in their morphology or the onset of symptoms. Three patients reported rapidly increasing size (Cases 4, 10, and 11). Two patients noted erosion or ulceration (Cases 10 and 13) and two patients noted spontaneously bleeding after incidental contact (Cases 7 and 9). The tumor was asymptomatic and only recognized during routine screening in four patients (Cases 1, 2, 5, and 8). 

### Tumor duration before diagnosis

The presence of the umbilical tumor prior to diagnosis was described in only 11 patients and ranged from 240 months (Case 5) to three months (Case 10), with a median of 24 months. The tumor had been present less than or equal to two years in 73% of patients (8/11) and had been present for six months or less in 27% of patients (3/11). Interestingly, one patient described a history of a slow-growing, red, scaly plaque around the umbilicus that was present for 20 years. It was originally diagnosed as eczematous dermatitis and treated with topical steroids and antibiotics with no resolution. After the lesion began bleeding spontaneously, BCC was diagnosed (Case 7).

### Location

BCCs typically arise on sun-exposed areas, such as the head and neck [19]. BCCs arising in the truncal region are not uncommon [4]. However, primary tumors of the umbilicus are very rare, and only 16 patients have been reported in the literature (Table 1). Metastatic tumors to the umbilicus (Sister Mary Joseph nodules) are much more common than primary tumors and are usually gastrointestinal adenocarcinomas [20].

### Morphology

Many of the umbilical BCCs presented as nodules (Cases 4, 6, 9, 10, 11, and 16); however, some were described as small papules (Cases 1 and 13) or plaques (Cases 2, 7, and 8). Some were described as asymmetric in appearance (Cases 2 and 10). The tumors were as small as 4 mm (Case 6) to as large as 2.7 x 1.5 cm (Case 7). One tumor demonstrated erosion (Case 10), one tumor demonstrated ulceration (Case 13), and two tumors demonstrated bleeding on contact (Cases 7 and 9).

The color of the tumor was described in 12 patients. It was flesh-colored in four patients (Cases 1, 6, 9, and 13) and red in one patient (Case 7). A pigmented BCC was clinically observed in seven patients (Cases 4, 6, 8, 10, 11, 15, and 16). This represented 88% (7/8 patients) of those with a diagnosis of pigmented BCC; the color of the lesion was not described in one patient whose pathology demonstrated pigmented BCC (Case 3).

### Pathology

The histologic subtype of umbilical BCC was described for 14 of the 17 tumors. All of the tumors had a histologic subtype that demonstrates less aggressive behavior [21]. Nodular histology was observed in 64% (9/14 carcinomas), including a patient whose tumor had mixed histology (Case 2). Three of the patients had a superficial BCC (Cases 2, 3, and 7).  

Previous studies have shown that BCCs in sun-sheltered regions predominantly are the superficial variant, while those in sun-exposed regions demonstrate a nodular histology [22]. In contrast, the majority of patients with umbilical BCC demonstrated nodular histology (Table 1).

A fibroepithelioma of Pinkus variant of BCC was also observed in two patients. A 67-year-old woman presented with a progressively enlarging dark brown to blue nodule over the umbilicus for three months. The patient had no conventional risk factors for BCC (Case 10). An 83-year-old man presented with two adjacent pink, polyploid masses arising from the umbilicus. The biopsy revealed two independent fibroepitheliomas of Pinkus tumors (Case 5). Fibroepithelioma of Pinkus subtype of BCC has a predilection for the trunk, in contrast to classic BCC, which arises in sun-exposed areas [23]. However, fibroepithelioma of Pinkus has also been described in other non-sun exposed sites, such as the axilla [5, 24-26].

Pigmented BCC, microscopically characterized by melanin in the tumor cells, the surrounding stroma, or both, was described in two of the patients with clinically pigmented lesions (Cases 5 and 8) and the one patient whose tumor color was not reported (Case 3). 

### Risk factors  

In addition to ultraviolet radiation exposure, risk factors for BCC include other environmental exposures, such as ionizing radiation, arsenic, asbestos, and dry cleaning solvents [27]. Other associations that may increase risk to develop BCC include trauma or chronic inflammation at the site, immunosuppression, genodermatoses (basal cell nevus syndrome (BCNS), Bazex syndrome, epidermolysis bullosa simplex, oculocutaneous albinism, Rombo syndrome, and xeroderma pigmentosum), history of prior BCC, and certain physical phenotypic characteristics (light color of eyes, hair, and skin) [5, 28-30]. None of the patients with umbilical BCCs had a history of extensive sun exposure to that region of their body. However, 31% (5/16) of patients with umbilical BCC had either an established or possibly associated tumor-related risk factor (Table 1).

A history of trauma and scarring may have contributed to the development of umbilical BCC in two patients. A 54-year-old woman underwent paraumbilical port placement for laparoscopic surgery, which resulted in an umbilical scar. BCC arising from the umbilical scar was diagnosed 21 years after the initial trauma (Case 9). The second patient, a 50-year-old African-American woman with BCNS, had several surgeries, including a ventral hernia repair four months earlier, at the site (Case 8). The mechanism of BCC arising from trauma remains elusive [14]. There have been at least nine reported cases of BCC arising from elective surgical scars, none of which involved the umbilicus [14].

Another patient had an epidermal inclusion cyst at the location of the tumor. Epidermal inclusion cyst is a benign epithelial lesion. Although it is not considered a risk factor for BCC, a few reports have described patients in whom a squamous cell carcinoma arose from an epidermal cyst [31-33]. Hence, the epidermal inclusion cyst may have represented a cutaneous immunocompromised district enabling the development of a BCC of the site [34]. 

Two patients had BCNS. BCNS is an autosomal dominant disorder that is associated with the development of multiple BCCs. In these individuals, BCCs usually develop in adolescence, with a median age of onset of 20 years [35]. The patients with BCNS and umbilical BCC developed their tumors at a younger age than the median age for all of the patients with BCCs at this location (49 versus 61 years). Similar to these individuals with BCNS and umbilical BCCs, BCCs have also been discovered in other sun-sheltered sites in patients with BCNS; these include the axilla, inguinal area, and buttocks [36-38].

### Pathophysiology

The majority of patients with umbilical BCC had no associated risk factors. Investigators have previously attempted to explain the pathogenesis of BCC arising in areas with no significant sun exposure. Strickland and colleagues postulated that ultraviolet radiation at distant sun-exposed sites may lead to a suppression of immune surveillance that in turn allows BCCs to develop in sun-protected sites [39]. Many have endorsed this theory as contributing to the formation of BCCs in other covered sites, such as the genital regions [40] and the axilla [25-26, 41]. Other researchers have suggested that local alterations in cell matrix interactions lead to changes in skin texture, shape, and tension that may contribute to tumor formation in sun-protected areas [42]. 

### Treatment

All of the patients with umbilical BCC had or shall have a surgical excision of the tumor. Mohs micrographic surgery (MMS) was performed for two patients (Cases 5 and 7). MMS is generally reserved for tumors located in areas of cosmetic concern or embryonal growth plates or both. However, MMS was justified in one patient because the tumor had invaded the umbilical stalk and there was a risk for seeding the peritoneum; the woman also received adjuvant radiation therapy because of the difficulty in monitoring recurrence in this anatomic area (Case 15). Similarly, MMS was performed in the second patient because the tumor was noted to extend deep into the umbilicus (Case 7). One patient with BCNS received carbon dioxide laser ablation in addition to surgery. The clinicians had successful experience with this combination treatment for plaques involving both concave and convex areas of the skin, such as those in the umbilicus, and purport that it may help reduce the surgical burden in BCNS (Case 2). 

### Prognosis

The prognosis for patients with umbilical BCC appears to be good after treatment. Follow-up was described for six patients and ranged from 12 months to 52 months. No recurrence or metastasis was reported for any of the patients (Table 1). 

## Conclusions

BCC appearing in the umbilicus is rare. Only 17 tumors in 16 patients have been described in the literature appearing at a median age of 61 years. Umbilical BCCs are 2.2 times more common in women than in men. In 80% of the patients, the tumor had been present for two or fewer years. The carcinoma typically presented as a nodule, papule, or plaque and the most common symptoms were ulceration and bleeding. The tumor was pigmented clinically or pathologically or both in eight patients. The most frequent histologic subtype was nodular; some of the patients had tumors that were fibroepithelioma of Pinkus, superficial, or mixed (nodular and superficial) histologic variant. Risk and/or possibly related factors that may be associated with BCC were observed in only 31% of patients (5/16); these included basal cell nevus syndrome, epidermal inclusion cyst, history of non-melanoma skin cancer, and previous surgical scar. The majority of patients with umbilical BCC had no tumor-related risk factors and did not have a history of prolonged sun exposure to the area. It has been postulated that decreased immune surveillance from ultraviolet exposure at other sites or local alterations in cell-matrix interactions or both may contribute to BCC in sites with little sun exposure, such as the umbilicus. Surgical management of the carcinomas was effective; the tumors were successfully treated by excision with or without examination of the margins during surgery. The prognosis of umbilical BCC is excellent; there have been neither recurrence nor metastasis following surgical excision of the neoplasm. 
